# EXERCISE TESTING IN ASSESSMENT AND MANAGEMENT OF PATIENTS IN CLINICAL PRACTICE - PRESENT SITUATION

**DOI:** 10.4103/0970-2113.59592

**Published:** 2008

**Authors:** Sumer S Choudhary, Sanjiw Choudhary

**Affiliations:** Department of Pulmonary Medicine, Sleep Medicine, Critical Care, Shree Ramjevan Choudhary Memorial Hospital and Research Centre, Nagpur, Maharashtra, India

**Keywords:** Exercise, heart, Interpretation, methodology, 6min walk test, testing

## OBJECTIVE

1. To review recent scientific advances in exercise testing methods and results that is important for a clinical practioner.
2. To understand the utility and limitations of different methods of exercise testing.
3. To understand appropriate method in assessment and management of patients.
4. To appreciate that exercise testing results can have greater clinical meaning when interpreted in context of relevant patient information.
5. To understand that additional study is required to further characterize both current and future roles of exercise testing in clinical medicine.

## INTRODUCTION

The need of the hour is to understand the different methods used worldwide to asses the patients exercise performance and response in clinical practice.

Clinical Exercise Testing (CET) is increasingly gaining importance in clinical medicine, by helping the clinician to objectively evaluate the physiological functions. The result helps to predict the outcome and mortality in different clinical circumstances.

## COMMON METHODS TO ASSES EXERCISE RESPONSE AND PERFORMANCES IN CLINICAL PRACTICE

Simple test are easily performed but limits physiological understanding.

More comprehensively performed tests may provide detail information and understanding but is costly and demanding. The clinician has to choose the type of test to perform for a particular patient

Commonly the following test is performed worldwide:-

1. 6 min walk test
2. Shuttle Walk Test
3. Exercise Induced Bronchoconstriction Test
4. Cardiac Stress Test
5. Clinical Exercise Test (CET)

## 6 MINUTE WALK TEST

It is a safe simple and practical test of sub maximal functional capacity, which measures the maximum distance walked by a subject in 6 minutes. Advantage of this test is that it provides an acceptable index of functional disability and correlates with oxygen uptake measured during comprehensive testing. This test gives very limited information regarding physiological contributors to activity related symptoms or about mechanism of exercise limitation. Currently this test is used in lung transplantation, lung volume reduction surgery, pulmonary rehabilitation and in predicting mortality in cardiac patients and patients with pulmonary vascular disorders.

## SHUTTLE WALK TEST

It measures the distance walked by a patient in a 10 meter course, being paced by an audio signals from a cassette. The intensity of exercise reached is comparable to test performed on a treadmill, as the walking speed is progressively increased until the patient reaches exhaustion. Modification of maximal SWT for determination of endurance performance – similar to maximal and constant (sub maximal) cycle ergometry may be done.

## EXERCISE INDUCED BRONCHOCONSTRICTION

In this physical activity triggers acute airway narrowing in patients with heightened airway responsiveness. In susceptible patients EIB typically occurs 5 to 10 minutes after exercise. and generally resolves in 20 to 30 minutes. In some clinical situation where bronchial challenge is unavailable or not diagnostic EIB should be undertaken.

Common protocols to be followed include exercise on treadmill or cycle ergometry at a workload of 60 %to 80% of predicted maximum or the intensity that will elicit a heart rate of 80% of predicted maximum for 6 to 8 minutes. The goal is to produce ventilation equal to those attained during activity to produce symptom of EIB.

15% percent decrease in FEV_1_ following exercise is diagnostic of EIB. And 10-15 % decrease in FEV_1_ would be suggestive of EIB.

## CARDIAC STRESS TEST

Common type of exercise testing, the primary purpose of which is diagnosis and management of myocardial infarction. Bruce protocol is commonly used and the single most reliable indication of ischemia is ST segment depression. During this test ECG and BP is measured, but the utility may be enhanced by concurrent measurement of ventilator parameters and respiratory gas exchange.

## CLINICAL EXERCISE TESTING (CET)

CET involves the measurement of respiratory gas exchange i.e. oxygen uptake, carbon dioxide, minute ventilation, other variables while monitoring ECG, blood pressure, pulse oximetry and exertion perceived (Borg Scale) during a maximal symptom limited incremental test on a cycle ergo meter or treadmill. Simultaneous measurement of blood gasses and spirometry provides with more detail information on gas exchange and ventilation. CET provides a global assessment of integrative exercise responses which are not adequately reflected by measurement of individual organ system function on rest. Peak oxygen uptake remains the gold standard for exercise capacity.

It has tradionaly been undertaken with an incremental stepwise or ramp control protocol to exhaustion. In patients of COPD, acute response to an inhaled bronchodilator was assessed using various exercise tests. The authors found endurance time with a constant – workload exercise (80% of maximal work rate)was the most responsive end point to the effect of bronchodilator showing 19% improvement in exercise duration time. Arterial blood gasses measured at 5 minute constant – work exercise testing may give practical and cost effective alternative when arterial oxygen saturation, PaO2, alveolar –arterial oxygen pressure difference and ratio of physiological dead space to tidal volume are required.

## INDICATIONS FOR EXERCISE TESTING IN CLINICAL PRACTICE

1. Evaluation of Exercise Intolerence
2. Evaluation of Unexplained exertional Dysponea
3. Evaluation of patients of cardiovascular diseases
4. Evaluation of Patients of respiratory diseases
  - COPD
  - ILD
  - Pulmonary Vascular Diseases
  - Cystic Fibrosis
5. Preoperative evaluation
6. Evaluation for transplantation and Lung Volume Reduction Surgeries
7. Pulmonary Rehabilitation
8. Impairment disability

Table 1 to 11 illustrates the indication, contraindication and guidelines laid down by various international authorities for cardio pulmonary exercise testing in clinical setting.

## CONCLUSION

Cardiopulmonary exercise test is a helpful tool for evaluation of the disease and management in clinical practice and rapidly evolving in one of the important investigative and diagnostic test. There are different methods used in various clinical setting. The clinical exercise testing a simple and easy to perform test for a pulmonologist as compared to the other conducted tests and relatively more simpler and cost effective test, which needs to be more frequently used in our day to day clinical practice in relevant patients.

**Table I:** Overview of Cardiopulmonary Exercise Testing

**Clinical Status Evaluation**
Clinical diagnosis and reason(s) for CPET
Health questionnaire (cardiopulmonary); physical activity profile
Medical and occupational history and physical examination
PFTs, CXR, ECG, and other appropriate laboratory tests.
Determination of indications and contraindications for CPET
↓
**Pretest Procedures**
Abstain from smoking for at least 8 h before the test
Refrain from exercise on the day of the test
Medications as instructed
Consent form
↓
**Conduct of CPET**
Laboratory procedures
Quality control
Equipment calibration
Protocol Selection
Incremental versus constant work rate; invasive versus
nominvasive
Patient preparation
Familiarization
12-lead ECG, pulse oximetry, blood pressure
Arterial line (if warranted)
Cardiopulmonary exercise testing
↓
**Interpretation of CPET Results**
Data processing
Quality and consistency of results
Comparison of results with approprate reference values
Integrative approach to interpretation CPET results
Preparation of CPET report

Definition of abbreviations : CPET = Cardiopulmonary exercise testing; CXR = chest X-ray; ECG; electrocardiogram; PFTs = pulmonary function tests.

**Table II:** Indications for Cardiopulmonary Exercise Testing

**Evaluation of exercise tolerance**
Determination of functional impairment or capacity (peak Vo_2_) Determination of exercise-limiting factors and pathophysiologic mechanisms.
**Evaluation of undiagnosed exercise intolerance**
Assessing contribution of cardiac and pulmonary etiology in coexisting disease. Symptoms disproportionate to resting pulmonary and cardiac tests. Unexplained dyspnea when initial cardiopulmonary testing is nondiagnostic.
**Evaluation of patients with cardiosvascular disease**
Functional evaluation and prognosis in patients with heart failure Selection for cardiac transplantation Exercise prescription and monitoring response to exercise training for cardiac rehabilitation. (special circumstance; i.e. pacemakers)
**Evaluation of patients with respiratory disease**
Functional impairement asessment (see specific clinical applications) Chronic obstructive pulmonary disease Establishing exercise limitation(s) and assessing other potential contributing factors, especially occult heart disease (ischemia) Determination of magnitude of hypoxemia and for O_2_ prescription When objective determination of therapeutic intervention is necessary and not adequately addressed by standard pulmonary function testing.
Interstitial lung diseases Detection of early (occult) gas exchange abnormalities Overall assessment/ monitoring of pulmonary gas exchange Determination of magnitude of hypoxemia and for O_2_ prescription Determination of potential exercise-limiting factors Documentation of therapeutic response to potentially toxic therapy Pulmonary vascular disease (careful risk-benefit analysis required) Cystic fibrosis Exercise-induced bronchospasm
**Specific clinical applications**
Preoperative evaluation Lung resectional surgery Elderly patients undergoing major abdominal surgery Lung volume resectional surgery for emphysema (currently investigational) Exercise evaluation and prescription for pulmonary rehabilitation Evaluation for impairment-disability Evaluation for lung, heart-lung transplantation Definition of abbreviations : Vo_2_ = oxygen consumption Reference 20

**Table III:** Absolute and Relative Contraindications for Cardiopulmonary Exercise Test

Absolute	Relative
Acute myocardial infarction (3-5 days)	Left main coronary stenosis or its equivalent
Unstable angina	Moderate stenotic valvular heart disease
Uncontrolled arrhythmias causing symptoms	Severe untreated arterial hypertension at rest
or hemodynamic compromise	(> 200 mm Hg systolic, > 120 mm Hg diastolic)
Syncope	Tachyarrhythmias or bradyarrhymias
Active endocardities	High-degree atrioventricular block
Acute myocarditis or pericarditis	Hypertrophic cardiomyopathy
Symptomatic severe aortic stenosis	Significant pulmonary hypertension
Uncontrolled heart failure	Advanced or complicated pregnancy
Acute pulmonary embolus or pulmonary infarction	Electrolyte abnormalities
Thrombosis of lower extremities	Orthopedic impairment that compromises exercise performance
Suspected dissecting aneurysm	
Uncontrolled asthma	
Pulmonary edema	
Room air desaturation at rest < 85%*	
Respiratory failure	
Acute noncardiopulmonary disorder that may affect exercise performance or be aggrevated by exercise (i.e. infection, renal failure, thyrotoxicosis)	
Mental impairment leading to inability to cooperate	

References 21, 22 and 23.

* Exercise patient with supplemental O_2_.

**Table IV:** Indications for Exercise Termination

Chest pain suggestive of ischemia
Ischemic ECG changes
Complex ectopy
Second or third degree heart block
Fall in systolic pressure > 20 mm Hg from the highest value during the test
Hypertension (> 250 mm Hg systolic; > 120 mm Hg diastolic)
Severe desaturation : Spo_2_ < 80% when accompanied by symptoms and signs of severe hypoxemia
Sudden pallor
Loss of coordination
Mental confusion
Dizziness or faintness
Signs of respiratory failure

Definition of abbreviations : ECG = electrocardiogram; Spo_2_ = arterial oxygen saturation as indicated by pulse oximetry. References 22, 24, 25 and 26.

**Table V:** Usual Cardiopulmonary Exercise Response Patterns

Measurement	Heart Failure	COPD	ILD	Pulmonary Vascular Disease	Obesity	Deconditioned
Vo_2_max or Vo_2_peak	Decreased	Decreased	Decreased	Decreased	Decreased for actual, normal for ideal weight	Decreased
Anaerobic threshold	Decreased	Normal/decreased indeterminate	Normal or decreased	Decreased	Normal	Normal or decreased
Peak HR	Variable, usually normal in mild	Decreased, normal in mild	Decreased	Normal/slightly decreased	Normal/slightly decreased	Normal/slightly decreased
O_2_ pulse	Decreased	Normal or decreased	Normal or increased	Normal	Normal or increased	Normal
(VE/MVV) × 100	Normal or decreased	Increased	Increased	Increased	Normal	Normal
VE/Vco_2_ (at AT)	Increased	Increased	Increased	Increased	Normal	Normal
VD/VT	Increased	Increased	Increased	Increased	Normal	Normal
Pao_2_	Normal	Variable	Decreased	Decreased	Normal/may increase	Normal
P(A-a)O_2_	Usually normal	Variably, usually increased	Increased	Increased	May decrease	Normal

Definition of abbreviations : AT = anaerobic threshold; COPD = chronic obstructrutive pulmonary disease; HR = heart rate; ILD = interstitial disease; MVV = maximal voluntary ventilation; P(A-a)O_2_ = alveolar-arterial difference for oxygen pressure; VD/VT = ratio of physiologic dead space to tidal volume; VE = minute ventilation; Vco_2_ = carbon dioxide output; Vo_2_ max = maximal oxygen uptake; Vo_2_ peak = peak oxygen uptake. References **37**, **38** and **28**

* Decreased, normal, and increased are with respect to the normal response.

**Table VI:** Measurements during Cardiopulmonary Exercise Testing

Measurements	Nominvasive	Invasive (Abgs)
External work	WR	
Metabolic gas exchange	Vo_2_, Vco_2_, RER, AT	Lactate
Cardiovascular	HR, ECG, BP, O_2_ pulse	
Ventilatory	Va, Vr, fR	
Pulmonary gas exchange	Spo_2_, Vr/Vco_2_, Vr/Vo_2_, PETO_2_, PETCO_2_	Pao^2^, Sao^2^, P(A-a)O_2_, VD/VT
Acid-base		pH, Paco^2^, standard HCO_3_
Symptoms	Dyspnea, fatigue, chest pain	

Definition of abbreviations : ABGs = Arterial blood gases; AT = anaerobic threshold; BP = Blood pressure; ECG = electrocardiogram; fR = respiratory frequency; HR = heart rate; P(A-a)O_2_ = alveolar-arterial difference for oxygen pressure; Paco_2_ = arterial carbon dioxide pressure; Pao_2_ = arterial oxygen pressure; PET-co_2_ = end-tidal Pco_2_; PETo_2_, = end-tidal Po_2_; RER = respiratory exchange ratio; Sao_2_ = arterial oxygen saturation; Spo_2_ = arterial oxygen saturation as indicated by pulse oximetry; Vco_2_ = carbon dioxide output; VE = minute ventilation; VD/VT = ratio of physiologic dead space to tidal volume; Vo_2_ = oxygen uptake; VT = tidal volume; WR = work rate. 31

**Table VII:** Suggested normal guidelines for interpretation of Cardiopulmonary Exercise Testing

Variables	Criteria of Normality
Vo_2_max or Vo_2_ peak	> 84% predicted
Anaerobic threshold	> 40% Vo_2_max predicted; wide range of normal (40-80%)
Heart rate (HR)	HRmax > 90% age predicted
Heart rate reserve (HRR)	HRR < 15 beats/min
Blood pressure	<220/90
O_2_ pulse (Vo_2_/HR)	> 80%
Ventilatory reserve (VR)	MVV - Vemax: > 11 or Vemax/MVV × 100 : < 85%.
	Wide normal range : 72 + 15%
Respiratory frequency (fR)	< 60 breaths/min
VE/ Vco_2_ (at AT)	< 34
VD/VT	< 0.28; < 0.30 for age > 40 years
Pao_2_ > 80 mm Hg	
P (A-a) O_2_	< 35 mm Hg

References 27, 28, 30, 35, 22 and 32

* Maximum or peak cardiopulmonary responses except for anaerobic threshold and VE/Vco_2_ at AT.

**Table VIII:** Integrative approach to the interpretation of Cardiopulmonary exercise testing results

1.	Determine reason(s) for CPET
2.	Review pertinent clinical and laboratory information (clinical status)
3.	Note overall quality of test, assessment of subject effort, and reasons for exercise cessation
4.	Identify key variables: initially Vo_2_, and then HR, VE, Sao_2_, and other measurements subsequently.
5.	Use tabular and graphic presentation of the data
6.	Pay attention to trending phenomena : submaximal through maximal responses.
7.	Compare exercise responses with appropriate reference values.
8.	Evaluate exercise limitation : physiologic versus nonphysiologic.
9.	Establish patterns of exercise responsess.
10.	Consider what conditions / clinical entities may be associated with these patterns.
11.	Correlae CPET results with clinical status.
12.	Generate CPET report.

Definition of abbreviations : CPET = cardiopulmonary exercise testing; HR = heart rate; Sao_2_ = arterial oxygen saturation; Ve = minute ventilation; Vo_2_ = oxygen uptake.

Reference 27

**Table IX:** Cardiopulmonary Exercise Response Patterns

Measurement	Heart Failure	COPD	ILD	Pulmonary Vascular Disease	Obesity	Deconditioned
Vo_2_max or Vo_2_peak	Decreased	Decreased	Decreased	Decreased	Decreased for actual, normal for ideal weight	Decreased
Anaerobic threshold	Decreased	Normal/decreased indeterminate	Normal or decreased	Decreased	Normal	Normal or decreased
Peak HR	Variable, usually normal in mild	Decreased, normal in mild	Decreased	Normal/slightly decreased	Normal/slightly decreased	Normal/slightly decreased
O_2_ pulse	Decreased	Normal or decreased	Normal or increased	Normal	Normal or increased	Normal
(VE/MVV) × 100	Normal or decreased	Increased	Increased	Increased	Normal	Normal
VE/Vco_2_ (at AT)	Increased	Increased	Increased	Increased	Normal	Normal
VD/VT	Increased	Increased	Increased	Increased	Normal	Normal
Pao_2_	Normal	Variable	Decreased	Decreased	Normal/may increase	Normal
P(A-a)O_2_	Usually normal	Variably, usually increased	Increased	Increased	may decrease	Normal

Definition of abbreviations : AT = anaerobic threshold; COPD = chronic obstructrutive pulmonary disease; HR = heart rate; ILD = interstitial disease; MVV = maximal voluntary ventilation; P(A-a)O_2_ = alveolar-arterial difference for oxygen pressure; VD/VT = ratio of physiologic dead space to tidal volume; VE = minute ventilation; Vco_2_ = carbon dioxide output; Vo_2_ max = maximal oxygen uptake; Vo_2_ peak = peak oxygen uptake. References 37, 36, 28

* Decreased, normal, and increased are with respect to the normal response.

**Table X:** 

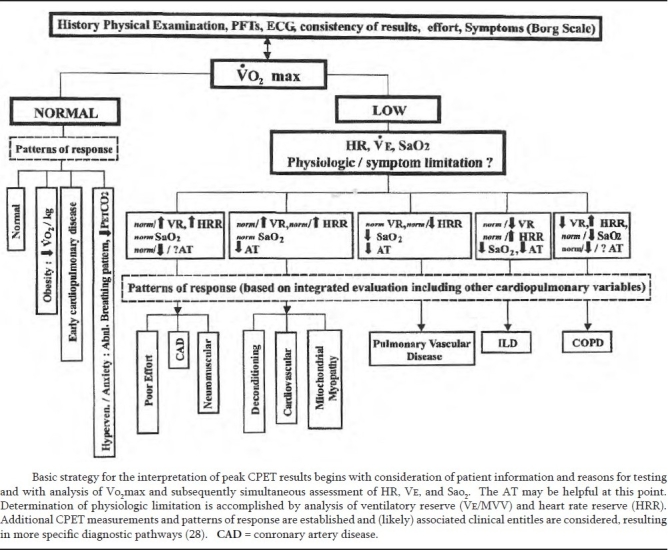

**Table XI:** Selected reference values for maximal incremental cycle exercise test

Variables	Equations*
Vo_2_, ml/min, male	W X [50.75 − 0.372 (A)]
Vo_2_, ml/min, female	(W − 43) × [22.78 − 0.17 (A)]
HR, beats/min	210 × 0.65 (A)*
O_2_ pulse, ml/beat	Predicated Vo_2_ max/predicted HRmax
Ve/MVV, %	˜ 72 + 15
AT, L/min (Vo_2_)	> 40% Vo_2_ pred

Definition of abbreviations : AT = Anaerobic threshold; HR = heart rate; Ve = minute ventilation; Vo_2_ = oxygen uptake.

Data from Referenes 32, 33 and 34

* Age (A) : years; height (H) : centimeters; weight (W), kilograms.

Predicted weight men : 0.79 × H − 60.7. Predicted weight women: 0.65 × H − 42.8. When actual weight > predicted, the predicted weight should be used in the equations. Wasserman and colleagues introduced new corrections factors (34, 28), which have not yet been published in peer reviewed journals.

^ See Lange-Andersen and coworkers (345).
